# Ultrasound-assisted solvent extraction of phenolics, flavonoids, and major triterpenoids from *Centella asiatica* leaves: A comparative study

**DOI:** 10.1016/j.ultsonch.2024.107091

**Published:** 2024-10-01

**Authors:** Tara Khursheed, Anees Ahmed Khalil, Muhammad Nadeem Akhtar, Ahood Khalid, Muhammad Rizwan Tariq, Tawfiq Alsulami, Robert Mugabi, Gulzar Ahmad Nayik

**Affiliations:** a University Institute of Diet and Nutritional Sciences, Faculty of Allied Health Sciences, The University of Lahore, 54000, Pakistan; b Department of Nutrition and Dietetics, National University of Medical Sciences (NUMS), Islamabad, Pakistan; c Department of Food Sciences, Faculty of Agricultural Sciences, University of the Punjab Lahore, Pakistan; d Department of Food Science & Nutrition, College of Food and Agricultural Sciences, King Saud University, Riyadh 11451, Saudi Arabia; e Department of Food Technology and Nutrition, Makerere University, Kampala, Uganda; f Marwadi University Research Centre, Department of Microbiology, Marwadi University, Rajkot, Gujarat 360003, India

**Keywords:** Ultrasonication, Bioactive compounds, Triterpenoids, Superoxide radical

## Abstract

*Centella asiatica* has been known for its significant medicinal properties due to abundance of bioactive constituents like triterpenoids and flavonoids. Nevertheless, an appropriate solvent system and extraction technique is still lacking to ensure optimized extraction of bioactive constituents present in *C. asiatica*. Recently, scientists are more focused towards application of green sustainable extraction techniques for the valuable components from plant matrix owing to their eco-friendly and safe nature. Among these, ultrasonication (US) is known as a valuable strategy for separation of bioactive components from medicinal plants. Hence, current research was performed to observe the effect of ultrasonication in the presence of five different solvents (Water, Hexane, Methanol, Chloroform, and Ethyl acetate) on total phenolic contents (TPC), total flavonoid contents (TFC), antioxidant properties (DPPH, ABTS, Nitric oxide radical activity, and Superoxide anion assay), and four major triterpenoid contents in *C. asiatica* leaves. Herein, ultrasound assisted methanolic extract (UAME) possessed maximum amount of TPC (129.54 mg GAE/g), TFC (308.31 mg QE/g), and antioxidant properties (DPPH: 82.21 % & FRAP: 45.98 µmol TE/g) followed by ultrasound-assisted Water extract (UAWE), ultrasound-assisted ethyl acetate extract (UAEAE), ultrasound-assisted n-hexane extract (UAHE), and ultrasound-assisted chloroform extract (UACE), respectively. Moreover, the superoxide radical and nitric oxide assays depicted a similar trend, revealing the highest percent inhibition for UAME (SO: 83.47 % & NO: 66.76 %) however, the lowest inhibition was displayed by UACE (63.22 % & 50.21 %), respectively. Highest content of major terpenoids were found in UAME of *C. asiatica* leaves as madecassoside (8.21 mg/g) followed by asiaticoside (7.82 mg/g), madecassic acid (4.44 mg/g), and asiatic acid (3.38 mg/g). Ultrasound-assisted extraction technique can be an efficient extraction method for bioactive compounds present in *C. asiatica*. However, ultrasonication along with methanol as an extraction solvent can surely enhance the extraction of valuable constituents. The results of this study provide an insight into major terpenoids, and antioxidants present in extracts of *C. asiatica*, implicating its use in ancient medicine systems and future drug development.

## 1 Introduction

Since ancient times, plants have been recognized by mankind for providing shelter, clothing, food, and medicinal resources. One of the most primitive human realizations was the encounter of medicinal plants throughout the civilizations. Medicinal plants have been utilized in different traditional medicine systems for the management of various ailments [1], [2], [3], [4]. Recently, research on herbal and medicinal plants has increased around the globe owing to their increased acceptability and significant potential in treating different chronic diseases [5]. Extracts of medicinal and herbal plants possess natural antioxidants that are most widely used as nutraceuticals in nearly 80 % of basic health care system of world’s population [6]. Medicinal plant extracts contain a cache of secondary constituents (phenolics, terpenoids, and alkaloids) having diverse biological properties. In recent times, scientists are focused on investigating the use of phytochemicals as a source of alternative medicines due to their origin, safe nature, easy accessibility, and availability [7].

*Centella asiatica* is a medicinal herb belonging to family *Apiaceae* and is commonly known as Brahmi, Gotu kola, Asiatic pennywort, and Indian penny wort. Natively, this herbaceous plant is found in swampy areas of tropical and temperate regions around the world specifically Asia [8]. Since ancient times, it has been prescribed by traditional practitioners for the treatment of stress, memory loss, inflammation, ulcer, anxiety, and bacterial infections [9]. Furthermore, it has also been used in Southeast Asian countries and traditional Chinese medications for prevention and/or management of various medical conditions like hysteria, rheumatism, syphilis, diarrhea, headache, asthma, and epilepsy [10]. Other than this, it is ingested as a green leafy vegetable having a mild pungent flavor and aroma [11]. *C. asiatica* encompasses diverse constituents such as vitamins, minerals, triterpenoids, flavonoids, and phenols [12]. This plant is known to be a rich source of antioxidants due to the presence of ample content of different flavonoids including naringin, rutin, kaempferol, apigenin, and quercetin [13].

According to different studies, therapeutic potential of *C. asiatica* and its biologically active phytochemicals like flavonoids, phenolics, and triterpenoids have been reported to have neuroprotective, cardioprotective, anti-inflammatory, and antioxidant properties. Bioactive compounds present in this plant are considered to alleviate various metabolic syndromes owing to their reduction of inflammation, preventing oxidation, and modifying the abnormalities of proteins related to mitochondria [13]. Phenolics and flavonoids occurring in leaves of *C. asiatica* have been found to possess a positive association among antioxidant and anticancer properties [14]. Extracts of *C. asiatica* have demonstrated significant antioxidant characteristics and have been evaluated using different assays [15]. Ameliorative potential of bioactive constituents present in extracts of *C. asiatica* against numerous ailments are due to their potential antioxidative characteristics [16].

Extraction is known as a vital first step for conducting bioactivity and other chemical analysis. Main purpose of extraction procedure is to gather maximum content of biologically active metabolites in resultant extract possessing highest antioxidative properties [17]. Since ancient times, several traditional techniques like maceration, percolation, infusion, and Soxhlet extraction have most widely been used for extraction of bioactive constituents from plants [18]. Nevertheless, these conventional methods have several limitations such as requirement of large quantity of solvent, enhanced extraction time, poor selectivity, low extraction yield, and labor intensive [19]. To overcome these limitations, nowadays, novel green extraction techniques like microwave-assisted extraction, ultrasonication, pulsed-electric field extraction, high-pressure extraction, and supercritical fluid extraction are being preferred over conventional techniques due to their efficient extraction yield, high selectivity, low extraction time, environment friendly and safe nature. Among these, ultrasonication is well-known among the scientific community as a viable technique for alternative to traditional procedures for extraction of biologically active constituents. Mainly, ultrasound-assisted extraction results in reduced extraction time, energy requirements, solvent utilization, and carbon dioxide emissions [20].

Antioxidant activity of any extract is directly associated to the type and amount of bioactive constituent being extracted in the extract. For the evaluation of antioxidant properties of any medicinal plant extract various assays like ABTS (2,2′-azino-bis(3-ethylbenzothiazoline-6-sulfonic acid), FRAP (ferric-reducing antioxidant power), and DPPH (2,2-diphenyl-1-picrylhydrazyl) are most widely performed [21]. Keeping in view the potential of ultrasonication in extraction of metabolites and importance of choice of solvent during extraction, current research was designed to assess the effect of ultrasound-assisted extraction in the presence of five different solvents (Chloroform, Hexane, Methanol, Ethyl acetate, and Water) on total phenolic contents (TPC), total flavonoid contents (TFC), antioxidant properties (DPPH, ABTS, Nitric oxide radical activity, and Superoxide anion assay), and major triterpenoid contents.

## 2 Materials and methods

### 2.1 Plant material

Matured *C. asiatica* leaves were collected from Ayub Agriculture Research Institute (AARI), Faisalabad, Pakistan in the month of March 2023. Prof. Dr. Rizwan Rasheed, Government College University Faisalabad, identified and authenticated the plant material. Voucher specimen number (# GC-BOT/114-2023) was placed in Herbarium of Department of Botany, Government College University Faisalabad, Punjab, Pakistan.

### 2.2 Chemicals

All the chemicals for determination of TFC (distilled water, NaNO_2_, NaOH, Quercetin), TPC (Folin-Ciocalteu reagent, Na_2_CO_3_, Gallic acid), DPPH-scavenging assay (2,2-diphenyl-1-picrylhydrazyl (DPPH), methanol), FRAP (TPTZ 98 %), acetate buffer, Trolox, HCl, FeCl_3_), Nitric oxide radical activity assay (Griess Illosvory reagent, napthyl ethylene diamine dihydrochloride, sodium nitroprusside, sulfanilic acid reagent), and superoxide anion assay (NADH, NBT, phenazine methosulfate) were procured from Sigma-Aldrich, Germany. For HPLC analysis, the standards namely madecassic acid, asiatic acid, madecassoside, amnd asiaticoside were purchased from Sigma-Aldrich (Germany).

### 2.3 Preparation of *C. Asiatica* leaf powder

The leaves of *C. asiatica* were washed using distilled water for the removal of dust and dirt particles. Later, they were dried in shade till a constant weight of leaves were achieved. After drying, the leaves were subjected to laboratory scale grinder for the collection of respective leaf powder.

### 2.4 Preparation of ultrasound assisted *C. Asiatica* leaf extract

The powder of *C. asiatica* leaves (2 gm) were subjected to ultrasound treatment using ultrasonicator (SB-600DTY, Ningbo Scientz Biotechnology Company Limited, Ningbo, China) with slight modification in methods adopted by Khalil et al. [22]. Purposely, each 2 g of leaf powder was mixed with 50 ml of five different solvents (Chloroform, Hexane, Methanol, Ethyl acetate, and Water), separately in a 100 ml flask. The general processing conditions of ultrasonicator were 40 kHz frequency, 25 °C temperature, and 15 min time. Theoretically, the energy input of the ultrasonicator was 250 W. For regulation of temperature of each sample, a water bath was used. After sonication, each sample was centrifuged at 25 °C and the resultant supernatants were filtered through polytetrafluoroethylene membrane fibers (0.22 µm). Afterwards, each representative extract was concentrated using rotary evaporator and stored at 4 °C till further analysis.

### 2.5 Determination of total phenolic contents (TPC)

The Folin Ciocalteu method was used for the determination of TPC in ultrasound assisted *C. asiatica* leaves extracts by adopting the protocols of Khalil et al. [23], [24]. For this purpose, each extract (125 µL) was initially added to distilled water (500 µL) followed by mixing with 125 µL Folin Ciocalteu reagent. This mixture was given a stay time of 5 min at ambient temperature. Afterwards, 125 µL of 7 % Na_2_CO_3_ was added in already prepared mixture. After preparation of the mixture, it was given a stay time of 90 min. In the end, the absorbance of each extract was evaluated at 765 nm through UV–Vis spectrophotometer. Each sample was taken as triplicate, and the TPC were expressed in mg (gallic acid equivalent (GAE)/gram).

### 2.6 Determination of total flavonoid contents (TFC)

TFC in ultrasound assisted *C. asiatica* leaves extracts were determined by following the method as adopted by Borrás-Enríquez et al. [25]. For this experimentation, each ultrasound assisted *C. asiatica* leaves extracts (100 µL) were individually added to 1325 µL of mixture A (5 % of NaNO_2_; 75 µL in distilled H_2_O; 1250 µL) and incubated for 5 min at room temperature. Later, 10 % AlCl_3_ (150 µL) were mixed and given a stay time of 5 min followed by addition of 440 µL of mixture B (1 M NaOH; 500 µL in distilled H_2_O; 425 µL). In the end, the absorbance of each extract was evaluated at 510 nm through UV–Visible spectrophotometer. Each sample was taken as triplicate, and the TFC were expressed in mg QE (quercetin equivalent)/gram.

### 2.7 Determination of antioxidant properties

#### 2.7.1 DPPH assay

DPPH radical scavenging activity was performed to assess the antioxidant properties of ultrasound assisted *C. asiatica* leaves extracts by slight modifications in methods of Ray et al. [26]. Purposely, herein this experimentation, 1000 µL of 0.1 mM DPPH prepared in methanol was thoroughly mixed with 1000 µL of each ultrasonically treated extract in the presence of different solvents (Chloroform, Hexane, Methanol, Ethyl acetate, and Water). Later, this mixture was incubated at room temperature for 30 min. Afterwards, the absorbance of each sample solution and blank solution were determined using UV–visible spectrophotometer. Percent inhibition in case of DPPH assay was determined through below mentioned formula:

$$\text{Percent inhibition (\textbackslash{}\%)}=\frac{\text{absorbance of blank - absorbance of each extract}}{\text{absorbance of blank}}\times \text{100}$$

#### 2.7.2 Ferric reducing antioxidant power (FRAP) assay

FRAP assay was accomplished to estimate the ferric reducing antioxidant power of ultrasound assisted *C. asiatica* leaves extracts by slight modifications in methods of Benzie and Strain et al. [27]. In this assay, the FRAP reagent comprised of 300 mM acetate buffer [sodium acetate trihydrate (3.1 g) & acetic acid (1.6 mL)], 2,4,6-Tripyridyl-S-triazine (TPTZ) solution (10 mM) prepared in hydrochloric acid (40 mM), and Iron (III) chloride hexahydrate (20 mM) with a 10:1:1 (v/v) volumetric ratio. The final concentration of all the extracts collected using ultrasonication in the presence of 5 different solvents, respectively, were 0.2 mg mL^−1^. Purposely, sample solution (400 μL) was mixed with FRAP reagent (3 mL). In the end the resultant mixture was placed in a water bath at 37 °C for 30 min. Further, the absorbance of all the extracts were determined by UV–Visible spectrophotometer at 593 nm. The standard used in FRAP assay was Trolox. Each sample was taken as triplicate, and the final results were expressed as micromoles Trolox equivalents/g.

#### 2.7.3 Nitric oxide scavenging (NOS) assay

Herein this assay, Griess Illosvory reaction was adopted to determine the nitric oxide radical inhibition assay of ultrasound assisted *C. asiatica* leaves extracts [28]. For this purpose, 500 µL of either standard solution or each extract (500 μg/mL) was incubated (2 h & 25 °C) with sodium nitroprusside (10 mM; 2000 µL) and saline phosphate buffer (500 µL) to form a total reaction mixture volume of 3000 µL. Afterwards, 1000 µL of sulfanilic acid reagent (0.33 % prepared in glacial acetic acid (20 %)) was mixed with 500 µL of already prepared reaction mixture and was held for 5 min followed by mixing of 1000 µL of napthyl ethylene diamine dihydrochloride. This solution was again given a stay time for 30 min at 25 °C. In the end the absorbance of both the extract sample and control was analyzed at 546 nm. Percent inhibition was determined using following formula:

$$\text{Percent inhibition (\textbackslash{}\%)}=\frac{\text{absorbance of blank - absorbance of each extract}}{\text{absorbance of blank}}\times \text{100}$$

#### 2.7.4 Super oxide anion (SOA) assay

In this assay, superoxide scavenging activity of ultrasound-assisted *C. asiatica* leaves extracts were determined by following the protocols as described by Fontana et al. [29]. For this assay, 1000 µL of reaction mixture comprised of 73 µM NADH, 15 µM Phenazine methosulphate solution, 20 mM phosphate buffer (pH: 7.4), 50 µM nitroblue tetrazolium solution, and 400 µg/mL of each ultrasound-assisted *C. asiatica* leaves extracts. The resultant solution was later incubated at 25 °C for 5 min and the absorbance was noted at 562 nm. The percent inhibition was calculated through following formula:

$$\text{Percent inhibition (\textbackslash{}\%)}=\frac{\text{absorbance of blank - absorbance of each extract}}{\text{absorbance of blank}}\times \text{100}$$

### 2.8 HPLC analysis

HPLC analysis was performed for the determination of four triterpenoids present in *C. asiatica* leaves extract collected by application of ultrasonication by following the protocols reported by Rafamantanana et al. [30]. For this, Waters 2690 HPLC system was used that comprised of an autoinjector, a pump, and a UV–Visible detector (2487 Waters, 206 nm). Separation was achieved through gradient elution using waters symmetry C18-column (I.D.: 150 × 4.6 mm & particle size: 5.0 µm). The mobile phase was water (A) and acetonitrile (B) having 1 mL min^−1^ flow rate. The detailed conditions for gradient elution were: 0 min, 80 % (A) & 20 % (B); 15 min, 65 % (A) & 35 % (B); 30 min, 35 % (A) & 65 % (B); and 45 min. 80 % (A) & 20 % (B). Stock solutions of madecassic acid, madecassoside, asiaticoside, and asiatic acid at three different concentrations ranging from 0.5 to 5 mg mL^−1^ concentrations were prepared in methanol and diluted for establishment of calibration curves.

### 2.9 Statistical analysis

Results were carried out in triplicate and expressed as mean ± S.D. One-way ANOVA was performed to assess the level of significance (*p* < 0.05) followed by Tukey’s HSD for comparison of means. Statistix software (ver. 8.1) was used to perform statistical analysis.

## 3 Results

### 3.1 Total phenolic contents, TFC, DPPH-scavenging activity, and ferric ion reducing antioxidant power

In this study, the TPC and TFC were assessed in the ultrasonically extracted phenolic compounds from *C. asiatica* leaves using different extraction solvents (Chloroform, Hexane, Methanol, Ethyl acetate, and Water). The results for the TPC and TFC revealed that ultrasonically assisted methanolic extract have the highest TPC and TFC as 129.54 ± 3.58 mg GAE/g and 308.31 ± 7.41 mg QE/g, respectively. Whereas the minimum content of TPC (21.09 ± 1.29 mg GAE/g) and TFC (128.82 ± 2.32 mg QE/g) were observed in ultrasonically extracted chloroform extract. Similar trends were noticed for DPPH radical scavenging activity and FRAP (ferric reducing antioxidant power). Evidently, Ultrasound-assisted extraction of *C. asiatica* leaf extract using methanol as solvent showed the values of 82.21 % and 45.98 µmol TE/g for DPPH and FRAP assays, respectively. However, the lowest DPPH and FRAP values were obtained for ultrasonically extracted chloroform extracts as 56.58 % and 27.01 µmol TE/g, respectively (Table 1).

**Table 1:** Antioxidant activity by different methods of ultrasonically extracted *C. asiatica* L. leaf extract using different solvents.

Parameters	TPC (mg GAE/g) Mean ± S.D.	TFC (mg QE/g) mean ± S.D.	DPPH (%) means ± S.D.	FRAP µmol TE/g Means ± S.D.
**Hexane**	30.54 ± 1.15^c^	133.72 ± 3.54^c^	60.91 ± 1.58^c^	28.98 ± 1.25^c^
**Chloroform**	21.09 ± 1.29 ^d^	128.82 ± 2.32^c^	56.58 ± 1.01^d^	27.01 ± 1.17^c^
**Ethyl acetate**	95.45 ± 2.02^b^	167.31 ± 4.02^b^	72.98 ± 1.61^b^	30.92 ± 1.14^c^
**Methanol**	129.54 ± 3.58 ^a^	308.31 ± 7.41 ^a^	82.21 ± 1.14^a^	45.98 ± 2.04 ^a^
**Water**	100.82 ± 1.94^b^	178.71 ± 4.25^b^	75.05 ± 1.21^b^	38.11 ± 1.68^b^

Results are expressed as means ± standard deviations (S.D.). Means in a column with same superscripts are not significantly different.

### 3.2 Superoxide anion radical and nitric oxide radical scavenging activity

**Fig. 1:**
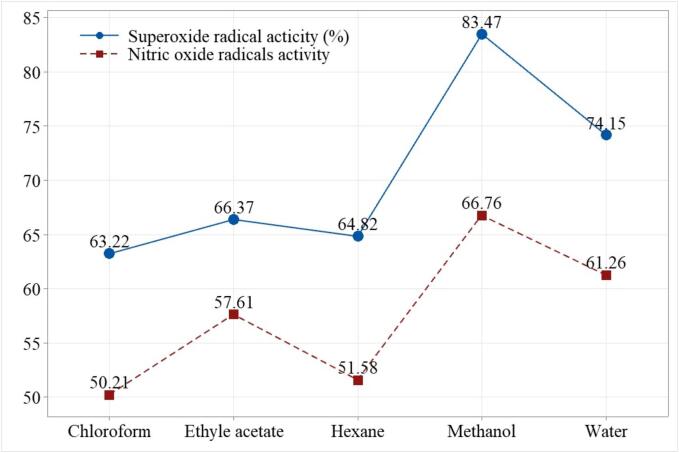
Superoxide anion radical activity (%) and nitric oxide radical scavenging activity (%) of ultrasonically extracted *C. asiatica* L. leaf extract using different solvents.

A similar trend was revealed for the superoxide anion (SO) and nitric oxide radical (NO) activity assays. The results obtained for both the assays are displayed in Fig. 1. The highest peaks (SO = 83.47 % and NO = 66.76 %) were observed for ultrasonically assisted methanolic extract of *Centella asiatica*. Followed by the peaks of ultrasonically assisted water extract displaying comparatively lower inhibition of 74.15 and 61.26 %. Further lower values were found for the ethyl acetate extract (66.37 ± 1.25 & 57.61 ± 1.16 %), hexane (64.82 ± 1.35 & 51.58 ± 1.58 %) and finally the lowest inhibition was for the ultrasonically assisted chloroform extract (63.22 ± 1.17 & 50.21 ± 1.35 %), respectively.

### 3.3 Major phytochemical compounds in *C. Asiatica* extracts

**Fig. 2:**
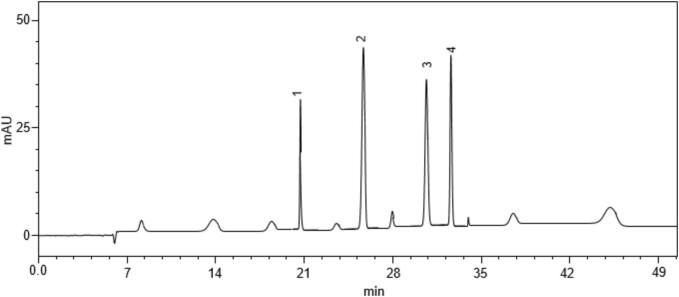
Standard chromatogram of Triterpenoids present in *C. asiatica* (**1.** Madecassoside, **2**. Asiaticoside, **3**. Madecassic acid, **4.** Asiatic acid).

In this study, 4 major compounds namely madecassoside, asiaticoside, madecassic acid, and asiatic acid were isolated through HPLC quantification. The Standard Chromatogram of triterpenoids present in *C. asiatica* is shown in Fig. 2. The quantities of these 4 compounds found in the *C. asiatica* ultrasonically assisted extracts obtained using different solvents (Chloroform, Hexane, Methanol, Ethyl acetate, and Water) are mentioned in Table 2. The values are expressed in mg/g and are presented as mean ± S.D. The capital letters depict the significance and concentration of the compounds obtained as compared to the other compounds.

**Table 2:** Four major triterpenoid contents (mg/g) in ultrasonically extracted *C. asiatica* L. leaf extract using different solvents.

Terpenoids	Treatments				
	Hexane (mg/g)	Chloroform (mg/g)	Ethyl acetate (mg/g)	Methanol (mg/g)	Water (mg/g)
**Madecassoside**	3.11 ± 0.11 ^d^	2.75 ± 0.13 ^d^	6.74 ± 0.22^c^	8.21 ± 0.12 ^a^	7.74 ± 0.15^b^
**Asiaticoside**	3.56 ± 0.13^c^	2.05 ± 0.14 ^d^	6.77 ± 0.18^b^	7.82 ± 0.11 ^a^	7.59 ± 0.21 ^a^
**Madecassic acid**	1.31 ± 0.08 ^d^	0.77 ± 0.08 ^e^	2.04 ± 0.09^c^	4.44 ± 0.14 ^a^	2.48 ± 0.11^b^
**Asiatic acid**	1.41 ± 0.07^c^	1.31 ± 0.03 ^d^	1.46 ± 0.08 ^bc^	3.38 ± 0.11 ^a^	1.66 ± 0.06^b^

Results are expressed as means ± standard deviations (S.D.). Means in a column with same superscripts are not significantly different.

As shown in Table 2, the concentrations of the different compounds for various solvent extracts of *C. asiatica* are expressed in mg/g of the quantity obtained. The highest concentration was obtained for all of the 4 compounds in ultrasonically assisted methanolic extract with values to be 8.21 ± 0.12 mg/g for madecassoside, 7.82 ± 0.11 mg/g for asiaticoside, 4.44 ± 0.14 mg/g for madecassic acid and 3.38 ± 0.11 mg/g for asiatic acid, respectively. Lower concentrations were obtained for ultrasonically assisted water extract in the range of 1.66 ± 0.06 to 7.74 ± 0.15 mg/g for all the compounds. Further lower quantity was obtained for the ultrasonically assisted ethyl acetate extract (1.46 ± 0.08 to 6.77 ± 0.18 mg/g), hexane (1.31 ± 0.08 to 3.56 ± 0.13 mg/g) and finally chloroform (0.77 ± 0.08 to 2.75 ± 0.13 mg/g), respectively. The capital letters show the comparison of each compound between different solvent extracts. Significant differences were observed among the concentrations of the same compound extracted with different solvents.

## 4 Discussions

In plants, the chemical composition of bioactive compounds in extracts varies depending upon their location in plant matrix, extraction technique used, and polarity of extracting solvent. Literature shows that various solvents and extraction methods have been employed for the extraction of biological active compounds from different parts of *C. asiatica.* Various studies have reported that unique triterpenes present in this plant such as madecassic acid, madecassoside, asiaticoside, and asiatic acid are considered responsible for the diverse bioactivity associated with this plant. These four triterpenoids present in different parts of *C. asiatica* plant are considered to be the primary and key bioactive metabolites. The content of plant phenolics, flavonoids, and terpenoids along with their associated bioactivity vary in reported literature owing to variation in extraction conditions, extraction techniques, type of solvent, and locality in plant matrix [31], [32], [33], [34]. The effectiveness of extraction process in terms of quantity and quality is dependent on various parameters such as solvent used, extraction procedure adopted, time, temperature, raw material particle size, and sample-to-solvent ratio. Evidently, numerous extracting methods are being used by scientists and industries for extraction of biomolecules from different parts of herbal and medicinal plants. Generally, Methanol is considered as the best choice for extracting solvent when used alone or in combination with some other solvent due to its polarity, solubility, evaporation rate, cost-efficiency, and compatibility [22], [23].

Ultrasonication is considered as one of the most widely used extraction method in food, cosmetic, and pharmaceutical industry for the extraction of biologically active phytochemicals from plant matrix [35]. In our study, we employed ultrasonication to *C. asiatica* leaves in the presence of five different solvents (Chloroform, Hexane, Methanol, Ethyl acetate, and Water) individually. Among all experimented solvents, ultrasound assisted methanolic extract (UAME) possessed maximum amount of TPC, TFC, and antioxidant properties followed by ultrasound-assisted Water extract (UAWE), ultrasound-assisted ethyl acetate extract (UAEAE), ultrasound-assisted n-hexane extract (UAHE), and ultrasound-assisted chloroform extract (UACE), respectively. Results of our study in case of TPC were in accordance with the findings of Kandasamy et al. [36]. They observed that TPC in *C. asiatica* extracts obtained by three different solvents *i.e.,* methanol, ethanol and cold water were in the range of 34.4 to 132.6 mg GAE/g. Similarly, methanolic extract of *C. asiatica* was also found to be rich in TFC as documented by Rashid et al. [37] that is in agreement with findings (TFC: 128.82-308.31 mg QE/g) of our current study. Moreover, Mustafa et al. [38] have also depicted similar results revealing that methanol as an extraction solvent resulted in efficient extraction of total phenolic and flavonoid contents. Likewise, results of four major triterpenes quantified in this research were also in accordance with earlier results of Seong et al. [39]. They reported the content of madecassoside, asiaticoside, madecassic acid and asiatic acid to be in range of 6.31–7.50 mg/g, 8.40–9.47 mg/g, 0.39–0.51 mg/g, and 1.64–1.83 mg/g, respectively. Madecassoside and asiaticoside, the two glycosides along with their subsequent aglycones (madecassic and asiatic acid) are considered as the predominant triterpenes responsible for their significant bioactivities [13]. In our current study we examined the variation in contents of four major triterpenes due to induction of ultrasonication in presence of different extraction solvents. Table 2 demonstrates the content of two glycosides and two corresponding aglycones and reveals the content of glycosides to be more as compared to aglycones. Maximum content of triterpenoid was noticed in glycosidic form in ultrasonicated methanolic extract of *C. asiatica* as madecassoside and asiaticoside followed by aglycone forms that were Madecassic acid and Asiatic acid. Additionally, results of our research were in harmony with previous investigation of Puttarak et al. [9], who were of the view that triterpenoids accumulate in leaves of *C. asiatica* in glycosidic form rather than aglycone form [9]. Conclusively, findings of our study support the fact that ultrasonication in presence of methanol may extract maximum triterpenoid contents from leaves of *C. asiatica.*

Numerous parameters like choice of solvent, extraction technique employed, time, and temperature influence the efficiency of extraction. Nowadays, green extraction techniques are being preferred over conventional extraction techniques owing to their efficient extraction yield, high selectivity, low extraction time, environment friendly and safe nature [19], [20]. Altered physical parameters like pressure, temperature, use of ultrasound and microwaves aid in effective rupturing of cell wall, therefore, enhancing the mass transfer of bioactive content to the extracting solvent. Choice of solvent and extraction technique are the two most critical parameters in an extraction process as solvent polarity and type of extraction method affects the extraction of class of bioactive constituents [20], [22]. Among all green extraction techniques, ultrasonication is being frequently used by both the scientific and industrial community due to its effectiveness, high selectivity, and rapid extraction time. In ultrasonication, acoustic cavitation phenomena effectively rupture the cell wall thereby enhancing the mass transfer of bioactive constituents into the extracting solvent [40]. Moreover, increased mass transfer results in shortening of extraction time along with less utilization of extracting solvent and decreased deterioration of heat-labile constituents present in plant matrix [41]. Our results are in agreement with outcomes of recent studies indicating the use of ultrasonication over conventional extraction as an effective method to enhance the extraction of biologically active components, therefore increasing the TPC, TFC, and antioxidant properties [42], [43]. Results in our study regarding antioxidant properties of *C. asiatica* extracts assessed through DPPH, FRAP, superoxide anion, and nitric oxide radical activity assays were higher as compared to the findings of Kumari et al. [44]. This increase in antioxidant properties may be due to application of ultrasounds, which effectively ruptured the cell wall and increased the diffusion of functional components into the extracting solvent. Enhanced concentration of polyphenols in extracting solvent resulted in increased antioxidant properties [45]. Likewise, Seong et al. [39] found that when extraction time, temperature and ultrasonic power are kept constant, the most effective factor enhancing the content of bioactive compound in extract is type of solvent. Therefore, in current study, methanol resulted in effective extraction as compared to other solvents due to its polarity, better solubility, more evaporation rate, cost-efficiency, and high compatibility [39].

Ultrasonication in presence of methanol as an extraction solvent was the most effective in terms of TPC, TFC, antioxidant properties, and content of four major triterpenoids. This was mainly due to ultrasonication that facilitated the disruption of cell wall and solubility of methanol, which in combination resulted in enhanced release of bioactive constituents [46]. Ultrasonication has been reported to be an effective extraction method for bioactive components from plant matrix as compared to other conventional techniques [47]. Conventional techniques are time-consuming that results in development of undesirable conditions, therefore causing reduction in antioxidant characteristics of extracts [48]. Among different extraction solvents, methanol has been reported as the most significant solvent being used for extraction of triterpenoids and phenolic constituents from *C. asiatica*. Variability in content of total phenolics, total flavonoids, and antioxidants among different extraction solvents may be due to the difference in polarity of solvents being used [49]. In general, literature highlights that increasing solvent polarity results in an increase in the extraction efficiency [50]. When it comes to polar solvents, hydrogen bond formation is being facilitated by dipole interactions that aid the increase in solubility of constituents. Even tough water is also a polar solvent but results in non-effective extraction of phenolic compounds from *C. asiatica* owing to the increased surface tension and rinses OH and COO-hydrophilic groups [50]. In our study, DPPH assay, FRAP assay, superoxide anion assay and nitric oxide radical activity assay revealed the maximum antioxidant potential in ultrasonicated methanolic extract followed by ultrasonicated water, ultrasonicated ethyl acetate, ultrasonicated chloroform, and ultrasonicated n-hexane extracts (Table 1). It is evident from literature that antioxidant potential of extracts is directly related to content of bioactive components present in each extract [18]. This direct association among antioxidant potential and bioactive compounds content is due to the reason that phenolic compounds show antioxidant properties by either scavenging, reducing, hydrogen donation, and metal chelation [12].

## 5 Conclusions

Conclusively, outcomes of our investigation signify the importance of ultrasound-assisted extraction technique as an effective method of extraction for collection of bioactive constituents present in *C. asiatica* leaves. Among five different experimented solvents, we reported that ultrasonication in presence of methanol resulted in maximum content of total phenolics, total flavonoids, along with significant antioxidant characteristics. Moreover, this remarkable antioxidative property in UAME may be due to presence of highest content of four major triterpenes (madecassoside, asiaticoside, madecassic acid, & asiatic acid), revealing its importance as medicinal plant in traditional medication and future drug development. In nutshell, our study illuminates the significance of using green and sustainable extraction techniques, to enhance the extraction of bioactive compounds from medicinal and herbal plants. Findings of our experimentation will contribute to better understanding of triterpenoids present in leaves of *C. asiatica*, strengthening the potential of this plant in traditional medication and future drug development activities.

## CRediT authorship contribution statement

**Tara Khursheed:** Writing – review & editing, Writing – original draft, Investigation, Formal analysis, Data curation, Conceptualization. **Anees Ahmed Khalil:** Writing – review & editing, Writing – original draft, Software, Methodology, Formal analysis, Data curation. **Muhammad Nadeem Akhtar:** Writing – review & editing, Software, Resources, Methodology, Formal analysis, Data curation. **Ahood Khalid:** Writing – review & editing, Software, Project administration, Investigation, Formal analysis, Conceptualization. **Muhammad Rizwan Tariq:** Writing – review & editing, Validation, Software, Methodology, Formal analysis, Data curation, Conceptualization. **Tawfiq Alsulami:** Writing – original draft, Supervision, Methodology, Investigation, Funding acquisition, Data curation. **Robert Mugabi:** Writing – review & editing, Resources, Methodology, Funding acquisition, Formal analysis, Data curation. **Gulzar Ahmad Nayik:** Writing – review & editing, Supervision, Resources, Methodology, Investigation, Formal analysis.

## Declaration of competing interest

The authors declare that they have no known competing financial interests or personal relationships that could have appeared to influence the work reported in this paper.
